# Identification of High-Risk Areas for the Spread of Highly Pathogenic Avian Influenza in Central Luzon, Philippines

**DOI:** 10.3390/vetsci7030107

**Published:** 2020-08-08

**Authors:** Roderick Salvador, Neil Tanquilut, Kannika Na Lampang, Warangkhana Chaisowwong, Dirk Pfeiffer, Veerasak Punyapornwithaya

**Affiliations:** 1 College of Veterinary Science and Medicine, Central Luzon State University, Science City of Muñoz 3120, Philippines; derek.salvador6@gmail.com; 2 Faculty of Veterinary Medicine, Chiang Mai University, Chiang Mai 50200, Thailand; 3 College of Veterinary Medicine, Pampanga State Agricultural University, Pampanga 2011, Philippines; neil@psau.edu.ph; 4 School of Environmental Science and Management, University of the Philippines, Los Banos 4030, Philippines; 5 Veterinary Public Health and Food Safety Centre for Asia Pacific (VPHCAP), Faculty of Veterinary Medicine Chiang Mai University, Chiang Mai 50200, Thailand; kannika.nalampang@cmu.ac.th (K.N.L.); warangkhana.chai@cmu.ac.th (W.C.); 6 Veterinary Epidemiology, Economics and Public Health Group, Royal Veterinary College, London AL9 7TA, UK; dirkpfeiffer@hotmail.com

**Keywords:** risk maps, transmission kernel, active surveillance, highly pathogenic avian influenza, Philippines

## Abstract

Highly pathogenic avian influenza virus (HPAIV) is a major problem in the poultry industry. It is highly contagious and is associated with a high mortality rate. The Philippines experienced an outbreak of avian influenza (AI) in 2017. As there is always a risk of re-emergence, efforts to manage disease outbreaks should be optimal. Linked to this is the need for an effective surveillance procedure to capture disease outbreaks at their early stage. Risk-based surveillance is the most effective and economical approach to outbreak management. This study evaluated the potential of commercial poultry farms in Central Luzon to transmit HPAI by calculating their respective reproductive ratios ($R_{0}$). The reproductive number for each farm is based on the spatial kernel and the infectious period. A risk map has been created based on the calculated R_0_. There were 882 (76.63%) farms with $R_{0}$ < 1. Farms with $R_{0}$ ≥ 1 were all located in Pampanga Province. These farms were concentrated in the towns of San Luis (*n* = 12) and Candaba (*n* = 257). This study demonstrates the utility of mapping farm-level $R_{0}$ estimates for informing HPAI risk management activities.

## 1. Introduction

Highly pathogenic avian influenza virus (HPAIV) has caused disease outbreaks among domestic poultry and wild bird populations, resulting in significant economic losses in many countries around the world. The potential of the infection to cause illnesses in humans is also a major concern. The Philippines confirmed an outbreak of HPAI H5N6 subtype, based on follow-up tests done in an Australian laboratory in August of 2017. Commercial poultry farms in barangays San Carlos and Santa Rita of San Luis, Pampanga were affected. After a week, cases were confirmed in Nueva Ecija, a province adjacent to Pampanga. A second wave of the outbreak was reported on 12 November 2017 in Cabiao, Nueva Ecija. A total of 23 farms were identified to have bird flu infection in the two provinces from July to November 2017. Sixteen farms were in Pampanga and seven in Nueva Ecija. Eight towns in Pampanga were affected (Apalit, Bacolor, Candaba, Lubao, Mabalacat, San Luis, San Simon, SantoTomas) and four towns in Nueva Ecija were included (Cabiao, Jaen, San Isidro, Zaragosa). During the outbreak, 15 layer chicken farms, 6 duck farms, and 2 quail farms were infected. An outbreak investigation identified key factors affecting the spread of the disease among farms including low biosecurity, having multiple species reared on the farm, no outbreak monitoring system, inability to identify the disease, and uncontrolled trade in poultry commodities. There were 45,682 birds that had died from the disease, while 393,515 birds were killed during the implementation of relevant outbreak management programs. The estimated daily loss as a consequence of the epidemic was about US$3.6 million [1]. Although increased mortality figures among poultry could not be definitively attributed to HPAI, there were accounts that many birds had died from symptoms consistent with HPAI several months prior to the confirmation of the outbreak.

Given the widespread under-reporting of this epidemic that has been associated with inadequate compensation and fear of trade restrictions, passive surveillance measures will be of limited effectiveness. Effective surveillance protocols enable authorities to detect disease outbreaks at an early stage, which should then trigger an immediate control response. As a result, the affected livestock industry will be prevented from incurring severe economic losses. While this may be the ideal situation, in most countries around the world the government’s veterinary service may not be able to cope with the necessary surveillance demands. This is due to the need for the simultaneous monitoring of many different diseases among various species and the need for a single agency to cover a large territory. Consequently, present surveillance strategies mainly rely on reporting by livestock keepers.

Implementation of an effective active surveillance protocol requires increased veterinary manpower, but most municipalities and cities are not prepared to invest in strengthening the capacity of their veterinary services. A more cost-effective alternative to active surveillance is a risk-based surveillance approach. By focusing on the high-risk strata of the population at risk, it is possible to conserve valuable resources. The identification of high-risk strata requires a good understanding of the epidemiological and socio-economic drivers of the spread of the relevant infectious disease. Risk factor studies have been used to identify spatial drivers of infection risk, and these have been used to generate risk maps [2,3,4,5]. Such outputs can be used to identify geographical areas where surveillance efforts should be implemented. Furthermore, several authors have described the successful use of a transmission kernel method for generating risk maps [6,7,8,9,10,11,12,13,14]. In brief, a transmission kernel represents the relative risk of transmission in terms of the distance to an infected farm. It is used as a proxy for all potential routes of infection that are spread between farms [7,12,15,16]. The method allows for the calculation of farm-level basic reproductive ratio values ($R_{0}$). The R_0_ is the expected number of secondary infections that occur among susceptible farms, as well as those that are caused by the first infectious farm throughout the initial infectious period [8,17]. A farm has the potential to cause an epidemic if its $R_{0}$ > 1, while it cannot if its $R_{0}$ < 1 [14]. The identification of farms that can serve as sources of epidemics can be set as the focus of the surveillance initiatives.

Highly pathogenic avian influenza is included in the priority list of diseases being monitored by the Philippine government. Therefore, this study evaluated the potential of commercial poultry farms in Central Luzon for transmitting HPAI by calculating their basic reproductive ratio numbers. This information was then used to produce a risk map based on the calculated R_0_.

## 2. Materials and Methods

### 2.1. Study Area

**Figure 1:**
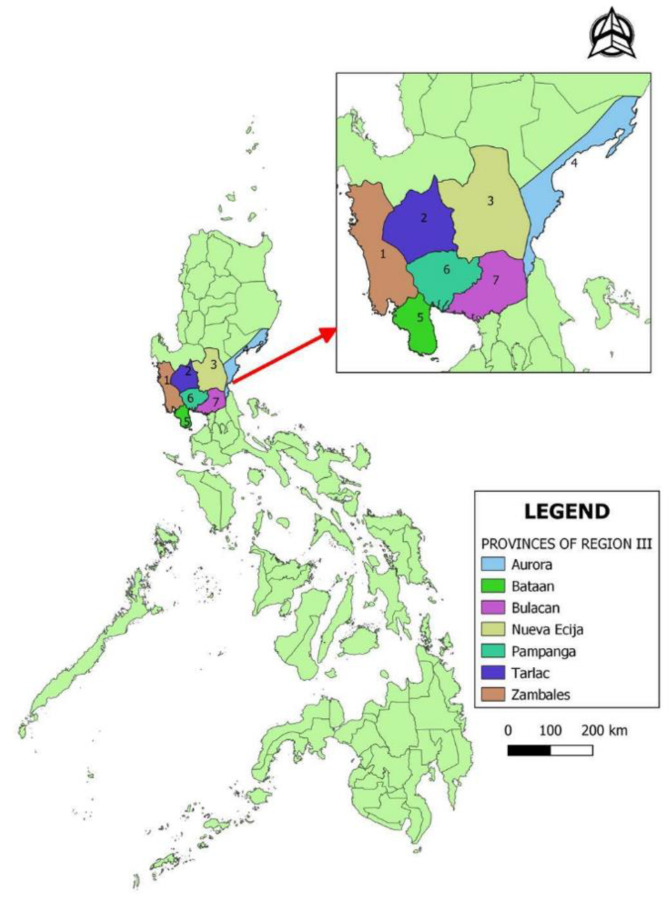
Map of the Philippines showing the study provinces.

The study was conducted in Central Luzon, Philippines. The Central Luzon region is composed of 7 provinces including Aurora, Bataan, Bulacan, Nueva Ecija, Pampanga, Tarlac, and Zambales (Figure 1). According to the data of the Philippine Statistics Authority, Central Luzon is the region with the highest density of ducks (32%) as of January 1, 2019 [18]. Moreover, the region is home to 68.8% of the total ducks kept in commercial farms. Likewise, the region is also home to the highest number of chickens with a 35.9% share of the country’s total chicken population over the same period of time [19]. A commercial farm is defined as a poultry operation with a minimum of 500 layers or 1000 broilers. For combined operation, a farm with at least 100 layers and 100 broilers is considered commercial.

### 2.2. Data Sources

HPAI outbreak data were provided by the Bureau of Animal Industry (BAI), Philippines. The poultry farms included in this study were based on the registration records for commercial poultry operations of the Provincial Veterinary Offices (PVO). The farm profiles (poultry population by species, farm coordinates) were compiled from farm visits that had been conducted throughout the region. Each farm’s maximum poultry capacity, and not the actual bird population at the time of the visits, were used in the analysis.

### 2.3. Model Formulation

The calculation of the farm level reproductive number ($R_{0}$) as described by Boender et al. [6,11] requires the computation of the transmission kernel (*h*) and the stochastic infectious period (T). The transmission kernel (*h*) was used to represent all forms of disease transmission between susceptible farms [15]. This parameter has been used to HPAI [7,20,21,22] and other infectious animal disease models like foot and mouth disease [15,23,24]. It refers to the infection hazard posed by an infectious animal from an infected farm to a susceptible animal of a susceptible farm. It is a function of the Euclidean distance (*r*) between farms. We adopted the kernel formulation (Equation (1)) from the studies of Boender et al. [6,11] and Dorigatti et al. [22]:

(1) $$h\left(r_{ij}\right)=\frac{h_{0}}{\left(1+{\left(r_{ij}/r_{0}\right)}^{\alpha }\right)}$$

The $r_{ij}$ refers to the Euclidean distance between an infectious farm *j* and a susceptible farm *i*. The $h_{0}$ is the maximum hazard rate, occurring when $r_{0}$= 0. The $r_{0}$ influences how far the hazard rate extends over distance. The *α* parameter influences the rate of decay in the hazard rate from the maximum. A dataset for the pairwise Euclidean distances (r) between farms was created. The distance between farms was calculated using the distGeo function of the *geosphere* in R statistical software version 3.5.3 (R Core Team, Vienna, Austria) [25].

To calculate the likelihood function, the force of infection on a susceptible farm *i* at time *t* was given by Equation (2) as described in [6]:

(2) $$\lambda_{i}(t)=\underset{j\neq i}{\sum }h(r_{ij})\;1\;\left[\;j\;\mathrm{is}\;\mathrm{infectious}\;\right]$$

With $\lambda_{i}\left(t\right)$ as the force of infection or the cumulative hazard rate experienced by farm *i* on day *t*, the probability that farm *i* is infected on day *t* is:

(3) $$qi\left(t\right)=1-e^{-\lambda i\left(t\right)}$$

The probability that farm *i* remains uninfected up to day *t* is:

(4) $$r_{i}\left(t\right)=e^{-}\sum_{s=1}^{t-1}\lambda_{i}\left(s\right)$$

Given the risk of infection above, the log-likelihood function is given by:

(5) $$\ell =-\underset{k\epsilon K}{\sum }\overset{t_{max}-1}{\underset{t=1}{\sum }}\lambda_{k}\left(t\right)-\underset{l\epsilon \Lambda }{\sum }\overset{t_{cul,l}-1}{\underset{t=1}{\sum }}\lambda_{l}\left(t\right)-\underset{m\epsilon M}{\sum }\overset{t_{inf,m}-1}{\underset{t=1}{\sum }}\lambda_{m}\left(t\right)\;+\underset{m\epsilon M}{\sum }log\left(1-e^{-\lambda_{m}\left(t_{inf,m}\right)}\right)$$

where the set K contains all farms that remained uninfected and that were not culled; Λ contains the farms that were not infected but that were culled (at times $t_{cul,\;l}$); M contains the farms that were infected (at times $t_{inf,\;m}$). With Equation (5), the estimates for the transmission kernel parameter values ($h_{0}$, $r_{0}$, and *α*) were calculated through maximum likelihood estimate (MLE) as described in [6] using the 2017 Philippines HPAI epidemic data. The *bbmle* functions of the R program [25] was used for this procedure.

Following the procedure described by Boender et al. [6], the basic reproduction number was estimated for each poultry farm in Central Luzon using the spatial transmission kernel ((*h*(*r_ij_*)) and the stochastic infectious period (T) of farms *i*. The reproduction number of farms *i*, $R_{0}$, is given by:

(6) $$R_{0}=\underset{j\neq i}{\sum }(1-E\;[e^{-h\left(r_{ij}\right)Ti}]$$

The computed R_0_ after 1000 iterations was used as the reference in developing the risk map. The *doParallel* functions of R [25] was used to calculate the reproductive ratio of each farm. Farms with at least 1.0 $R_{0}$ were classified as high-risk farms. The risk map was created using QGIS (Open Source Geospatial Foundation Project, Zurich, Switzerland) [26].

## 3. Assumptions

Upon infection, a farm passes through a latency period of 1–2 days [22,27,28,29]. In this stage, the avian influenza virus develops within the host until the host becomes infectious by shedding the virus. Viral inoculation experiments of HPAIV showed that birds can start shedding the virus after 0.76 day [30], or 1.1 day [31]. Though it is possible that birds can start to become infectious in less than 24 h after infection, we adopted the results of Vander Goot [32]. Their results showed that a latent period of 1–2 days fits the data better than the model with a latent period of less than one day. This is likewise adopted by Boender et al. [6], and Dorigatti et al. [22] in their HPAI simulation studies. The detection of HPAI in the farm is assumed to be on days 5–7. This is based on mortality thresholds [33]. Several studies have shown mortality thresholds to be the most reliable indicator of AI infection in poultry farms [34,35]. The assumptions used here are not specific for the H5N6 subtype. However, the range of transmission rate of the HPAI H5N6 virus in domestic poultry is close to or within the range of transmission rates found for other HPAI subtypes. This is generally applicable in cases where transmission experiments are limited. The infection experiment by Jiao (2016) showed that inoculated chicken died at 4–7 days post inoculation (dpi) [36]. Chicken succumbed by 4–5 dpi based on the results of Kim (2018) [37]. We assumed that suspicion of HPAIV infection due to increased mortalities will happen between 5 and 7 days after infection. Based on the response of farm managers to a survey, they will be notifying authorities about the presence of the disease 5–7 days post detection. Using the outbreak data as reference, culling of an infected farm is initiated 1–3 days after receipt of the report. The days required for completion of culling of infected farms after reporting were 1–3 days, depending on the size of the farm. Specifically, for farms with a population ≤50,000, the culling needs 1 day; for farms with 50,000 < population ≤100,000, the culling needs 2 days; for farms with population >100,000, the culling needs 3 days. The infectious period lasts until the completion of culling.

## 4. Results

### 4.1. Farm Location

**Figure 2:**
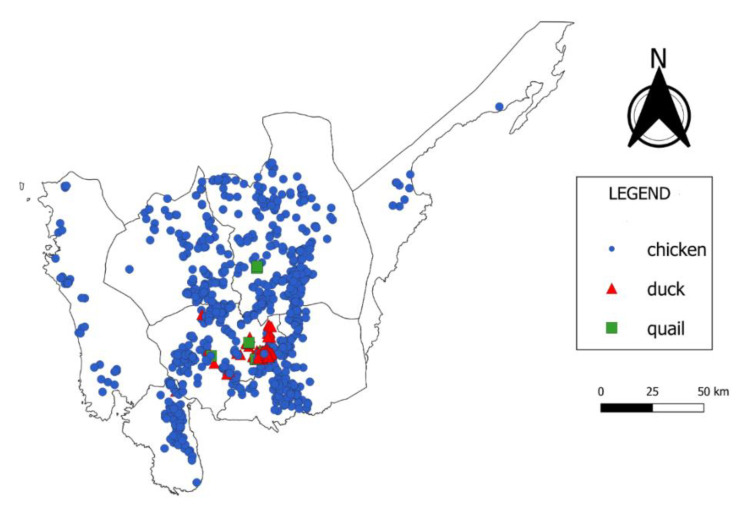
Map of Central Luzon, Philippines showing the physical location of all 1151 commercial poultry farms. Chicken farms are represented by blue dots. Quail farms are represented by green squares. Duck farms are represented by red triangles.

The locations of the poultry farms included in this study are shown in Figure 2. There was a total of 1151 poultry farms that kept chickens (*n* = 880), ducks (*n* = 260), or quail (*n* = 11). The duck farms identified in the study were all located in Pampanga Province. The poultry farm distribution data in the study area, and their average flock size are presented in Table 1.

Poultry production levels are considered high in the provinces of Nueva Ecija, Pampanga, and Bulacan. These provinces represent 78.8% of the total poultry farms in the region. Likewise, they share 70.3% of the total poultry population. Large scale operations were observed in the provinces of Bulacan, Tarlac, Nueva Ecija, and Zambales considering their high average flock sizes. In contrast, Pampanga was able to significantly contribute to the regional poultry production levels because of the high number of poultry farms (*n* = 485) despite its lower average flock size. Aurora had the smallest number of farms. This may be due to the logistical challenges of poultry farming that exist in the area. This would likely be a consequence of its remoteness and it being in a mountainous area.

### 4.2. Farm Practices

At this study site, ducks were raised mainly for the purposes of egg production. Incorporated into the management protocol of duck farms is the concept of foraging. This provides ducks the opportunity to consume snails which are believed to improve the shell quality of the eggs produced by these ducks. Foraging sites include swamps and empty rice paddies. This practice requires frequent movement of the flock in search of foraging areas. Ducks were moved via trucks with customized battery cages. Foraging in a particular geographical area may range from 3 to 7 days before the ducks are moved to a different area. Fresh and processed eggs were then sold to traders or directly to consumers through bakeries and restaurants.

In contrast, chickens are always kept within the boundaries of the farm. Chicken raising operations can be dedicated either to layer or broiler chickens. At the end of a production cycle, poultry manure can be sold to vegetable farms as fertilizer. Broilers were either bought by traders or by the company partner if the operation was under contract. For the layers, the spent hens were sold to poultry traders.

### 4.3. Transmission Parameters

Maximum likelihood estimation based on the 23 infected farms during the 2017 HPAI outbreak resulted in the following parameter estimates: $h_{0}$ = 0.0012 day^−1^ (CI: 0.0001–0.1), $r_{0}$ = 3.4 km (CI: 1.001–10.0), and α = 1.4 (CI: 1.001–5.0), with ℓ=−206.99 and AIC = 419.98.

### 4.4. Farm-Level Basic Reproductive Ratio Values

The summary statistics for the farm-level farm reproductive ratio are presented in Table 2. The median of the farm-level basic reproductive ratio values for the 1151 commercial farms in Central Luzon was 0.424. There were 882 (76.63%) farms with a R_0_ of less than 1. Chickens are the predominant species (96.2%, 849/882) raised in these farms. Meanwhile, ducks are the major species (86.98%, 234/269) in farms with a $R_{0}$ ≥ 1.

**Figure 3:**
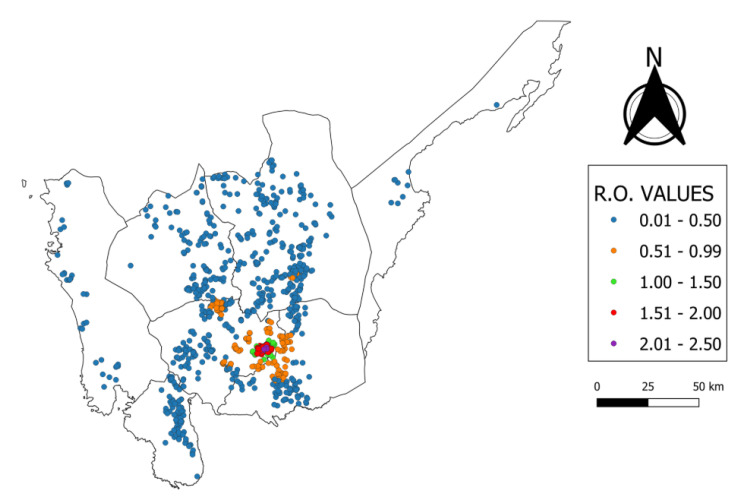
Risk map of highly pathogenic avian influenza transmission in Central Luzon, Philippines.

Notably, around 77% (882/1151) of the total poultry farms in Central Luzon are outside the high-risk areas. These farms include 26 duck farms, 7 quail farms, and 849 chicken farms (Figure 3). All farms with a $R_{0}$ ≥ 1 are located in Pampanga Province. These farms are concentrated in the towns of San Luis (*n* = 12) and Candaba (*n* = 257). This area is composed of 234 duck farms, 31 chicken farms, and 4 quail farms.

## 5. Discussion

In this study, farm-level basic reproductive ratio values were calculated and used to generate a risk map for the transmission of HPAI in Central Luzon. The transmission kernel was derived from the profiles of the 23 farms infected during the 2017 HPAI epidemic in the Philippines. These farms were identified through reports from farm owners to the BAI. It is acknowledged that the results from this modeling exercise need to be used cautiously, since only this single outbreak could be used for generating the transmission kernel and there may have been underreporting of farm locations and disease events. The findings are discussed below in the context of the wider literature to allow an assessment of their epidemiological validity.

To construct the model, the factors used to account for the species-specific transmissibility were developed by Hayama [8], Keeling [10], and Dorigatti [22]. The number of animals in the farm is likewise added to the equation used by Keeling [10] and Hayama [23]. The inclusion of a species-specific transmission coefficient is considered important if there are variations in susceptibility and the occurrence of infections among different species, such as is the case with avian influenza. Unfortunately, no further parameters could be derived from this small dataset. The approach used here has also been applied in order to generate risk maps for FMD [11] and HPAI [6] in the Netherlands.

Moreover, with the available dataset, kernel density estimation may be the best approach. Other spatial techniques include the cluster detection method, the relative space method, and risk factor identification [38]. The research studies of Ahmed et al. [39], Loth et al. [2], and Tiensin et al. [40] likewise were aimed at identifying high-risk areas for the spread of HPAI. Both Ahmed and Loth used data acquired from the Bangladesh outbreaks, while Tiensin et al. [40] used data obtained from the Thailand outbreaks. Bangladesh had two epidemic waves of HPAI in two consecutive years, while Thailand generated outbreak data from 2004 to 2007. In these cases, the availability of large and more detailed datasets allowed for the application of the spatial cluster detection method and risk factor identification.

The calculated $R_{0}$ can be used to inform risk-based surveillance. Currently, the Philippines does not have laws providing veterinary authorities with the legal powers to investigate the health status of animal farms in the absence of a disease outbreak. But if high risk farms can be identified based on scientific criteria, such as the R_0_, it might be possible to convince farmers of the need for risk-based surveillance protocols. Large poultry industry companies could use the spatial patterns of R_0_ to identify low-risk geographical areas to target in their production expansion initiatives.

The results indicate that duck farms represent the majority (87%) of farms with an $R_{0}$ ≥ 1 (Table 2). Wild and domestic ducks have been associated with the spread and maintenance of avian influenza viruses (AIV) [40,41,42]. Free grazing ducks played a significant role in the maintenance and transmission of infections during the second wave of HPAI epidemic of Thailand in 2004 [43] The AIV of the H5N6, H5N2, and H9N3 subtypes were isolated from healthy ducks for sale in live markets in Hanoi, Vietnam [44]. The study of Sturm-Ramirez et al. (2005) showed that the HPAI H5N1 virus causes minimal signs of diseases in ducks. Consequently, they can spread HPAI silently and efficiently among domestic and wild birds in Asia [45].

Part of the production management procedure for duck farmers involves grazing in post-harvest rice paddy fields, and thereby, requires frequent movement between habitat sites which the ducks share with other wild birds. Analysis of the HPAI-H5N1 epidemic in Thailand revealed that outbreaks are commonly reported in the central and northern regions where duck population densities are high [46]. Similarly, low pathogenic avian influenza (LPAI) subtypes H4N6 and H3N8 were isolated from sentinel flocks of ducks in Thailand several years after the epidemic [47]. The 2017 HPAI H5N6 outbreak in the Philippines confirmed that HPAI is virulent among domestic ducks. This was also observed among H5N1 infected ducks in 2002. The virus killed many aquatic bird species. Earlier, ducks infected with highly pathogenic H5 or H7 avian influenza virus are consistently asymptomatic or had very mild disease. The outbreaks in Thailand, Vietnam, and Japan from 2002 to 2004 were pathogenic to ducks. Hulse-Post et al. [48] reported that there is a possibility for AIV to revert to its non-pathogenic form among the duck population. Their study showed a trend toward decreased pathogenicity among ducks. The virus remained highly pathogenic to chickens and humans. This finding demonstrates the limitation of using only clinical signs to determine infection with HPAI among the duck population. Consequently, the reliance on passive surveillance will not be effective. Thus, regular monitoring through testing among the duck population should be included in the strategy against HPAIV.

The vast majority of farms with $R_{0}$ ≥ 1 were located in Candaba, Pampanga (95.5%, *n* = 257). The Candaba swamp that is in this town is a recognized stopover for migratory birds from the Northern Hemisphere, especially for birds from China and Russia. During the dry season, part of the swamp is utilized for rice production [49]. Epidemiological studies of HPAI outbreaks have identified the relevance of the proximity to natural wetlands and water bodies as an important risk factor [50,51,52,53]. These areas provide an interface for contact between domestic and wild ducks.

The identification of the risk factors associated with the spread of HPAI is outside the objectives of this study. The spatial risks presented above are brought out as the observed features of the farms that are at high risk for outbreaks (high farm density, high duck farm density, and proximity to the Candaba swamp). The risks that were identified agree with the findings of prior studies. Programs to address these spatial risks should be integrated into the plans being put into place to address the avian influenza concern. The potential indirect contact between domestic ducks and infected migratory birds through sharing the same foraging areas cannot be avoided. Feeding in post-harvest rice paddy fields is integral to the production system of duck farms. Surveillance efforts should be comprehensive among the duck population, and both low and highly pathogenic AIV should be covered. To supplement active surveillance practices, sentinel flocks can be set up to monitor the presence of AIV.

The risk of spread is more likely to be observed in areas with high levels of farm density. The affected area in the 2010 FMD epidemic in Japan was the Miyazaki Prefecture. This prefecture is home to the highest density of cattle farms (165.0 farms/100 km^2^) and the second highest density of pig farms (9.8 farms/100 km^2^). Proximity (≤1.5 km) to an infected farm has been associated with the spread of HPAI in Italy [53]. The HPAI outbreaks in Thailand occurred in areas with high levels of poultry farm-density [46].

In part, the density of farms can be regulated through the development of policies that limit the number of farms allowed in an area. This can be implemented in current low-risk areas. Regulations, however, can only cover commercial farms as these are required to obtain government permits for their operation. The establishment of small-scale operations cannot be controlled unless a mandatory registration of farms with at least 50 birds is implemented. To address the risk of outbreaks spreading into high-density areas, farms must be required to maintain high levels of biosecurity. There should be information campaigns on improved biosecurity practices among poultry raisers in both high- and low-risk areas. Another aspect of the programs to be instituted would include the identification and description of the trading dynamics that exist between the farms located in high-risk areas and their customers. This information will facilitate contact-tracing in the event of an outbreak. With the risk map as a guide, management strategies in the event of an HPAI outbreak are dependent upon the location of the index farm. Quarantine procedures and the culling of infected farms located outside high-risk areas would be sufficient in controlling the potential spread. In contrast, farms with a high probability of disease transmission require additional pre-emptive culling to mitigate the possibility of an outbreak. One limitation of this study is the unknown number of unregistered quail and duck farms in contrast to poultry farms, which by law have been officially registered. While 261 duck farms were registered, we believe that there were other commercial duck farms outside Pampanga. Poultry farms were identified based on the records of Provincial Veterinary Offices. Apparently, a number of small-scale duck and quail producers were not compliant in registering their operations. Compared to the operation of other species, a commercial quail farm does not require much space, and the transportation of quail eggs to the market can be done in concealed ways such as in vans or in the boots of cars. Quails are known to be highly susceptible to HPAI, and the clinical disease period can last longer than in chickens. Thus, quail farms are known to have high potential for transmitting the disease [54,55]. It is therefore likely that the current map is biased due to missing observations that are associated with specific farm species that are known to be important risk factors for avian influenza.

Based on the findings from this study, it is recommended to expand the coverage of the risk map to a national scale. Thus, mandatory registration of all poultry holdings will be required. This will then potentially allow to identify further high-risk areas where more intensive surveillance should be implemented. Furthermore, a national database of poultry farm profiles would allow epidemic modeling projects related to other avian diseases.

## 6. Conclusions

In conclusion, the HPAI risk map was generated by using the transmission kernel estimated from the 2017 epidemic data. This method allowed us to identify high-risk areas by calculating the farm-level basic reproductive ratio. The resulting risk map facilitates visual communication of the findings to decision-makers, and it thereby serves as an effective tool in support of the development of HPAI control and prevention strategies for specific geographic areas. Importantly, risk maps are dynamic, and need to be critically reviewed and updated once new information becomes available. Extending the risk mapping process to the national level with inclusion of small-scale poultry farms (of at least 50 birds) may allow identification of other high-risk areas in the country.

## Figures and Tables

**Table 1:** Distribution of poultry farms in the provinces of Central Luzon.

Province	Poultry Farms	Average Population
Aurora	9	8222
Bataan	90	54,147
Bulacan	154	92,858
Nueva Ecija	268	57,823
Pampanga	485	27,257
Tarlac	112	87,231
Zambales	33	104,164

**Table 2:** Summary of farm-level basic reproductive ratio values.

R_0_	Ducks	Quail	Chickens	Total
0.01–0.50	8	6	705	719
0.51–0.99	18	1	144	163
1.00–1.50	8	3	12	23
1.51–2.00	135	0	15	150
2.01–2.5	91	1	4	96

## Data Availability

The data used in this study is available at figshare. DOI: https://doi.org/10.6084/m9.figshare.11987604.v1. The geographical coordinates of farms used in the analysis were slightly modified due to privacy issue.
